# Di­chlorido­[6,8,22,24,34,36-hexa­methyl-33,35-diaza-3,11,19,27-tetra­azonia­penta­cyclo[27.3.1.1^5,9^.1^13,17^.1^21,25^]hexa­triaconta-1(33),5,7,9(34),13,15,17(35),21,23,25(36),29,31-dodeca­ene-κ^6^ *N* ^3^,*N* ^11^,*N* ^19^,*N* ^27^,*N* ^33^,*N* ^35^]dipalladium(II) bis(per­chlor­ate) *N*,*N*-di­methyl­formamide disolvate methanol disolvate

**DOI:** 10.1107/S1600536813022666

**Published:** 2013-08-23

**Authors:** Kohei Oda, Yasuhiro Funahashi, Hideki Masuda

**Affiliations:** a Department of Frontier Materials, Graduate School of Engineering, Nagoya Institute of Technology, Gokiso-cho, Showa-ku, Nagoya 466-8555, Japan; b Department of Chemistry, Graduate School of Science, Osaka University, Machikaneyama-cho, Toyonaka, Osaka 560-0043, Japan; c PRESTO, Japan Science and Technology Agency (JST), Japan

## Abstract

In the crystal structure of the title compound, [Pd_2_(C_36_H_42_N_6_)Cl_2_](ClO_4_)_2_·2C_3_H_7_NO·2CH_3_OH, the dinuclear Pd^II^ complex cation lies on an inversion center. Each Pd^II^ ion has a distorted square-planar coordination sphere, defined by three N atoms of the macrocyclic ligand and a chloride ion. The Pd^II^ complex cations and the methanol mol­ecules are linked through N—H⋯O and O—H⋯O hydrogen bonds, forming a zigzag chain along [101]. An intra­molecular N—H⋯Cl hydrogen bond is also observed.

## Related literature

For palladium(II) complexes with 2,6-bis­(amino­meth­yl)pyridine, see: Arnáiz *et al.* (2002 ▶). For dipalladium(II) complexes having a Pd^II^–Cl unit, see: Suess & Peters (2010 ▶); Goforth *et al.* (2013 ▶). For palladium(II) complexes containing a macrocyclic ligand, see: Parker (1985 ▶); Parker *et al.* (1985 ▶). For a similar macrocyclic ligand, see: Allmendinger *et al.* (2003 ▶). For a similar cryptand ligand, see: Higa *et al.* (2010 ▶).
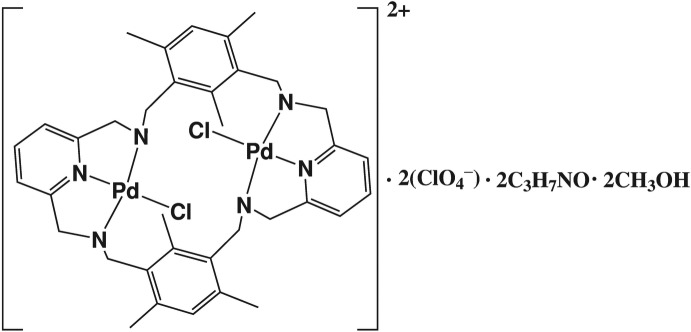


## Experimental

### 

#### Crystal data

[Pd_2_(C_36_H_42_N_6_)Cl_2_](ClO_4_)_2_·2C_3_H_7_NO·2CH_4_O
*M* *_r_* = 1255.68
Monoclinic, 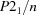

*a* = 10.917 (2) Å
*b* = 19.083 (4) Å
*c* = 12.705 (3) Å
β = 104.201 (2)°
*V* = 2566.0 (8) Å^3^
*Z* = 2
Mo *K*α radiation
μ = 0.98 mm^−1^
*T* = 173 K
0.20 × 0.20 × 0.20 mm

#### Data collection

Rigaku Mercury70 diffractometer
Absorption correction: numerical (*NUMABS*; Rigaku, 1999 ▶) *T*_min_ = 0.751, *T*_max_ = 0.823
19797 measured reflections
5826 independent reflections
4980 reflections with *F* ^2^ > 2σ(*F* ^2^)
*R*_int_ = 0.029

#### Refinement

*R*[*F* ^2^ > 2σ(*F* ^2^)] = 0.038
*wR*(*F* ^2^) = 0.097
*S* = 1.08
5826 reflections
324 parameters
H atoms treated by a mixture of independent and constrained refinement
Δρ_max_ = 0.80 e Å^−3^
Δρ_min_ = −0.81 e Å^−3^

### 

Data collection: *CrystalClear* (Rigaku, 2001 ▶); cell refinement: *CrystalClear*; data reduction: *CrystalClear*; program(s) used to solve structure: *SIR92* (Altomare *et al.*, 1993 ▶); program(s) used to refine structure: *SHELXL97* (Sheldrick, 2008 ▶); molecular graphics: *CrystalStructure* (Rigaku, 2001 ▶); software used to prepare material for publication: *CrystalStructure*.

## Supplementary Material

Crystal structure: contains datablock(s) global, I. DOI: 10.1107/S1600536813022666/is5294sup1.cif

Structure factors: contains datablock(s) I. DOI: 10.1107/S1600536813022666/is5294Isup2.hkl

Additional supplementary materials:  crystallographic information; 3D view; checkCIF report

## Figures and Tables

**Table 1:** Selected bond lengths (Å)

Pd1—Cl1	2.3084 (9)
Pd1—N1	2.062 (3)
Pd1—N2	1.942 (3)
Pd1—N3	2.087 (3)

**Table 2:** Hydrogen-bond geometry (Å, °)

*D*—H⋯*A*	*D*—H	H⋯*A*	*D*⋯*A*	*D*—H⋯*A*
O6—H18⋯O6^i^	0.84	2.33	2.757 (5)	112
N1—H12⋯O6	0.78 (4)	2.21 (4)	2.930 (5)	153 (4)
N3—H13⋯Cl1^ii^	0.71 (4)	2.67 (5)	3.332 (4)	156 (4)

Symmetry codes: (i) 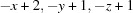; (ii) 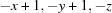.

**Fig. 1.**
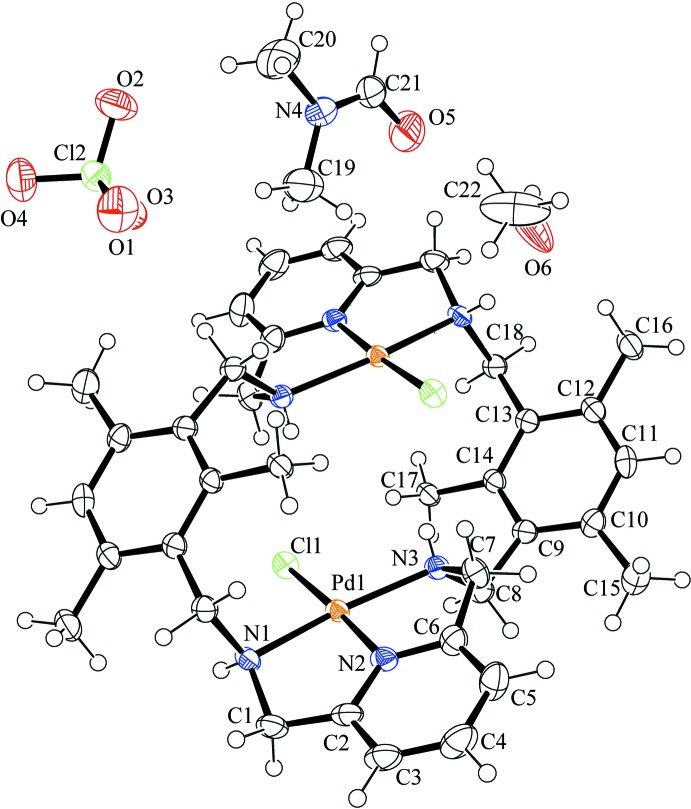
The view of molecular structure of the title compound with the atomic numbering scheme. Displacement ellipsoids are drawn at the 50% probability level.

**Fig. 2.**
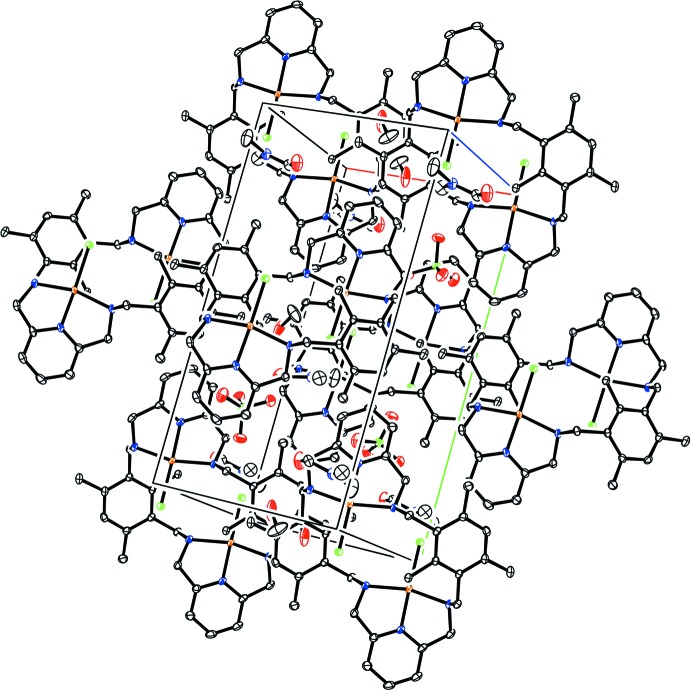
A partial packing diagram of the title compound. H atoms are omitted for clarity.

## supplementary crystallographic information

### 1. Comment

2,6-Pyridinedicarboxaldehyde and 2,4-bis-aminomethyl-1,3,5-trimethyl benzene were used for synthesis of an imine macrocycle, and the highly selective cyclization reaction resulted in a [2+2] stoichiometry, in a similar procedure to a previous study (Allmendinger *et al.*, 2003). It can be facilely reduced by NaBH_4_ to give the corresponding amine analogue containing two 2,6-di(aminomethyl)pyridine units. The spacer unit can capture two transition metal ions inside the macrocyclic ligand molecule. Previously, similar macrocyclic ligands containing dinuclear palladium(II) complexes have been synthesized (Parker *et al.*, 1985). Using the amine macrocyclic ligand, we have fortunately prepared a novel dinuclear palladium(II) complex, and succeeded in elucidating the crystal structure. The single-crystal X-ray diffraction analysis has revealed that the title compound, [Pd^II^_2_(C_36_H_42_N_6_)Cl_2_](ClO_4_)_2_.2DMF.2CH_3_OH, has crystallized in the monoclinic space group *P*2_1_/n, and the unit cell contains two palladium(II) ions and two chloride ions inside the macrocyclic ligand. The structure of the dinuclear palladium(II) complex with the macrocyclic ligand has a center of symmetry inside the ligand molecule. The two perchlorate anions, two crystalline DMF and two methanol molecules are located outside the ligand sphere (Fig. 2). The palladium(II) center has a four-coordinate square-planar geometry occupied by three N atoms of the macrocyclic ligand and one chloride ion (Fig. 1).

### 2. Experimental

The macrocyclic ligand (C_36_H_42_N_6_.0.5H_2_O) was synthesized by the following method. 2,4-Bis(aminomethyl)-1,3,5-trimethylbenzene (2.67 g, 1.50 × 10 ^-2^ mol) was dissolved in methanol (200 ml). To this solution, a methanol solution (200 ml) of 2,6-pyridinedicarboxaldehyde (2.05 g, 1.52 × 10 ^-2^ mol) was added dropwise. The mixed solution immediately gave a white precipitate, which was collected by vacuum filtration, washed with water (100 ml), and dried under vacuum. The white precipitate was dissolved in dichloromethane (300 ml), and an ethanol solution (300 ml) of NaBH_4_ (2.59 g, 6.84 × 10 ^-2^ mol) was added in portion. After the reaction mixture was stirred for 6 h, the solution was acidified with 0.1 N HCl aq. and alkalified with 1 N KOH aq. Evaporation of the resulting solution under reduced pressure gave a white precipitate of the macrocyclic ligand, which was filtered out, washed with water, and dried under vacuum (yield 3.74 g, 88%). Analysis, calculated for C_36_H_42_N_6_.0.5H_2_O: C 76.22, H 8.26, N 14.81; found: C 76.20, H 8.24, N 14.86. ^1^H NMR (300 MHz, CDCl_3_*versus* TMS, δ, p.p.m.): 1.88 (*s*, 4H, NH), 2.36 (*s*, 12H, PhCH_3_), 2.37 (*s*, 6H, PhCH_3_), 3.74 (*s*, 8H, CH_2_), 3.95 (*s*, 8H, CH_2_), 6.86 (*s*, 2H, Ph), 7.14 (*d*, 4H, pyridine), 7.58 (*t*, 2H, pyridine).

The dipalladium(II) complex was synthesized by the following procedure. The macrocyclic ligand (56.7 mg, 1.00 × 10 ^-4^ mol) was dissolved in methanol–chloroform (7 ml, 1:1 *v*/*v*). To this solution, a solution of (Et_4_N)_2_[Pd^II^Cl_4_] (105.4 mg, 2.00 × 10 ^-4^ mol) in methanol–chloroform (7 ml, 1:1 *v*/*v*) was added. After the mixed solution was stirred for 6 h at room temperature, a saturated methanol solution (3 ml) of (*n*-Bu)_4_NClO_4_ was added to give a bright-yellow precipitate as a product. It was filtered out, washed with diethyl ether, and dried under vacuum condition. Single crystals suitable for X-ray crystallographic analysis were obtained by recrystallization from *N*,*N*-dimethylformamide–methanol–dimethyl ether.

### 3. Refinement

H atoms attached to C atoms were positioned geometrically and treated as riding, with aromatic C—H = 0.95 Å, methyl C—H = 0.98 Å and methylenic C—H = 0.99 Å, and with *U*_iso_(H) = 1.2*U*_eq_(C). The hydroxyl H atom was positioned geometrically and treated as riding, with O—H = 0.84 Å and with *U*_iso_(H) = 1.5*U*_eq_(O). H atoms on N atoms were located in a difference Fourier map and isotropically refined.

### Crystal data

**Table:**

[Pd_2_(C_36_H_42_N_6_)Cl_2_](ClO_4_)_2_·2C_3_H_7_NO·2CH_4_O	*F*(000) = 1288.00
*M**_r_* = 1255.68	*D*_x_ = 1.625 Mg m^−^^3^
Monoclinic, *P*2_1_/*n*	Mo *K*α radiation, λ = 0.71070 Å
Hall symbol: -P 2yn	Cell parameters from 6824 reflections
*a* = 10.917 (2) Å	θ = 3.0–27.5°
*b* = 19.083 (4) Å	µ = 0.98 mm^−^^1^
*c* = 12.705 (3) Å	*T* = 173 K
β = 104.201 (2)°	Block, yellow
*V* = 2566.0 (8) Å^3^	0.20 × 0.20 × 0.20 mm
*Z* = 2	

### Data collection

**Table:**

Rigaku Mercury70 diffractometer	4980 reflections with *F*^2^ > 2σ(*F*^2^)
Detector resolution: 7.314 pixels mm^-1^	*R*_int_ = 0.029
ω scans	θ_max_ = 27.5°
Absorption correction: numerical (*NUMABS*; Rigaku, 1999)	*h* = −13→13
*T*_min_ = 0.751, *T*_max_ = 0.823	*k* = −24→24
19797 measured reflections	*l* = −16→16
5826 independent reflections	

### Refinement

**Table:**

Refinement on *F*^2^	Secondary atom site location: difference Fourier map
*R*[*F*^2^ > 2σ(*F*^2^)] = 0.038	Hydrogen site location: inferred from neighbouring sites
*wR*(*F*^2^) = 0.097	H atoms treated by a mixture of independent and constrained refinement
*S* = 1.08	*w* = 1/[σ^2^(*F*_o_^2^) + (0.0439*P*)^2^ + 3.481*P*] where *P* = (*F*_o_^2^ + 2*F*_c_^2^)/3
5826 reflections	(Δ/σ)_max_ = 0.001
324 parameters	Δρ_max_ = 0.80 e Å^−^^3^
0 restraints	Δρ_min_ = −0.81 e Å^−^^3^
Primary atom site location: structure-invariant direct methods	

### Special details

**Table:**

Refinement. Refinement was performed using all reflections. The weighted *R*-factor (*wR*) and goodness of fit (*S*) are based on *F*^2^. *R*-factor (gt) are based on *F*. The threshold expression of *F*^2^ > 2.0 σ(*F*^2^) is used only for calculating *R*-factor (gt).

### Fractional atomic coordinates and isotropic or equivalent isotropic displacement parameters (Å^2^)

**Table:**

	*x*	*y*	*z*	*U*_iso_*/*U*_eq_	
Pd1	0.63072 (2)	0.578980 (12)	0.164247 (18)	0.02062 (8)	
Cl1	0.61781 (7)	0.45840 (4)	0.17120 (6)	0.02688 (16)	
Cl2	0.21267 (8)	0.73945 (4)	0.37351 (7)	0.03456 (19)	
O1	0.1928 (3)	0.66584 (15)	0.3537 (3)	0.0598 (9)	
O2	0.2752 (3)	0.75349 (19)	0.4847 (3)	0.0614 (9)	
O3	0.2891 (3)	0.76593 (18)	0.3048 (3)	0.0579 (9)	
O4	0.0927 (3)	0.77435 (16)	0.3469 (3)	0.0582 (9)	
O5	0.7672 (3)	0.6378 (3)	0.5140 (3)	0.0713 (11)	
O6	0.8813 (3)	0.5059 (3)	0.4312 (4)	0.0974 (17)	
N1	0.8226 (3)	0.58758 (14)	0.2298 (2)	0.0236 (6)	
N2	0.6424 (3)	0.68049 (14)	0.1703 (2)	0.0244 (6)	
N3	0.4436 (3)	0.59993 (14)	0.0836 (3)	0.0233 (6)	
N4	0.5730 (3)	0.62401 (17)	0.5422 (3)	0.0401 (7)	
C1	0.8448 (3)	0.65695 (18)	0.2889 (3)	0.0324 (8)	
C2	0.7484 (3)	0.70951 (18)	0.2337 (3)	0.0284 (7)	
C3	0.7542 (4)	0.78140 (19)	0.2459 (3)	0.0370 (8)	
C4	0.6500 (4)	0.8209 (2)	0.1954 (4)	0.0432 (9)	
C5	0.5432 (4)	0.78952 (19)	0.1295 (4)	0.0394 (9)	
C6	0.5423 (3)	0.71802 (17)	0.1165 (3)	0.0287 (7)	
C7	0.4453 (3)	0.67333 (17)	0.0408 (3)	0.0287 (7)	
C8	0.3352 (3)	0.58744 (17)	0.1373 (3)	0.0254 (7)	
C9	0.2169 (3)	0.56955 (16)	0.0501 (3)	0.0239 (6)	
C10	0.1281 (3)	0.62113 (16)	0.0033 (3)	0.0266 (7)	
C11	0.0349 (3)	0.60461 (17)	−0.0898 (3)	0.0290 (7)	
C12	0.0270 (3)	0.53965 (17)	−0.1397 (3)	0.0253 (7)	
C13	0.1117 (3)	0.48650 (16)	−0.0896 (3)	0.0227 (6)	
C14	0.2032 (3)	0.50048 (16)	0.0067 (3)	0.0223 (6)	
C15	0.1251 (4)	0.69419 (18)	0.0475 (4)	0.0397 (9)	
C16	−0.0711 (4)	0.5274 (2)	−0.2446 (3)	0.0351 (8)	
C17	0.2865 (3)	0.44240 (16)	0.0660 (3)	0.0263 (7)	
C18	0.1021 (3)	0.41548 (16)	−0.1441 (3)	0.0250 (7)	
C19	0.5132 (5)	0.6064 (4)	0.4304 (5)	0.085 (2)	
C20	0.4934 (5)	0.6278 (3)	0.6182 (4)	0.0630 (13)	
C21	0.6937 (4)	0.6389 (2)	0.5736 (4)	0.0413 (9)	
C22	0.8039 (7)	0.4662 (4)	0.4680 (5)	0.093 (3)	
H1A	0.8393	0.6505	0.3649	0.0388*	
H1B	0.9306	0.6743	0.2899	0.0388*	
H2	0.8282	0.8033	0.2881	0.0444*	
H3	0.6514	0.8702	0.2059	0.0518*	
H4	0.4724	0.8170	0.0942	0.0473*	
H5A	0.3607	0.6947	0.0317	0.0344*	
H5B	0.4646	0.6718	−0.0313	0.0344*	
H6A	0.3565	0.5484	0.1899	0.0305*	
H6B	0.3205	0.6300	0.1769	0.0305*	
H7A	0.1585	0.7273	0.0025	0.0476*	
H7B	0.1770	0.6960	0.1223	0.0476*	
H7C	0.0379	0.7069	0.0465	0.0476*	
H8	−0.0256	0.6394	−0.1204	0.0348*	
H9A	−0.1268	0.4889	−0.2349	0.0422*	
H9B	−0.0290	0.5151	−0.3019	0.0422*	
H9C	−0.1211	0.5701	−0.2650	0.0422*	
H10A	0.0123	0.4052	−0.1784	0.0300*	
H10B	0.1337	0.3791	−0.0886	0.0300*	
H11A	0.3743	0.4511	0.0637	0.0315*	
H11B	0.2586	0.3974	0.0311	0.0315*	
H11C	0.2808	0.4410	0.1418	0.0315*	
H14A	0.5533	0.5645	0.4093	0.1019*	
H14B	0.5227	0.6456	0.3831	0.1019*	
H14C	0.4232	0.5972	0.4232	0.1019*	
H15A	0.4566	0.5816	0.6244	0.0756*	
H15B	0.4255	0.6619	0.5920	0.0756*	
H15C	0.5442	0.6425	0.6895	0.0756*	
H16	0.7268	0.6513	0.6475	0.0496*	
H17A	0.7712	0.4922	0.5217	0.1116*	
H17B	0.8488	0.4243	0.5020	0.1116*	
H17C	0.7334	0.4521	0.4077	0.1116*	
H18	0.9179	0.5336	0.4803	0.1461*	
H13	0.440 (4)	0.5775 (19)	0.038 (4)	0.022 (10)*	
H12	0.849 (4)	0.5575 (19)	0.271 (3)	0.022 (9)*	

### Atomic displacement parameters (Å^2^)

**Table:**

	*U*^11^	*U*^22^	*U*^33^	*U*^12^	*U*^13^	*U*^23^
Pd1	0.01775 (13)	0.02451 (13)	0.01903 (12)	−0.00282 (9)	0.00344 (8)	−0.00189 (8)
Cl1	0.0265 (4)	0.0257 (4)	0.0279 (4)	−0.0018 (3)	0.0055 (3)	0.0035 (3)
Cl2	0.0329 (5)	0.0345 (5)	0.0355 (5)	−0.0029 (4)	0.0068 (4)	0.0010 (4)
O1	0.066 (2)	0.0311 (15)	0.083 (3)	−0.0037 (14)	0.0182 (18)	0.0012 (15)
O2	0.059 (2)	0.086 (3)	0.0343 (16)	−0.0044 (18)	0.0023 (14)	−0.0027 (16)
O3	0.0519 (18)	0.077 (3)	0.0452 (17)	−0.0205 (16)	0.0132 (15)	0.0059 (15)
O4	0.0382 (16)	0.0521 (18)	0.079 (3)	0.0083 (14)	0.0032 (16)	−0.0072 (16)
O5	0.0459 (19)	0.112 (4)	0.062 (3)	0.0133 (19)	0.0262 (17)	0.007 (2)
O6	0.0401 (18)	0.163 (5)	0.082 (3)	−0.009 (3)	0.0015 (18)	0.086 (3)
N1	0.0194 (13)	0.0306 (14)	0.0187 (13)	−0.0018 (11)	0.0007 (11)	0.0003 (11)
N2	0.0196 (13)	0.0286 (13)	0.0250 (13)	−0.0061 (11)	0.0055 (11)	−0.0062 (11)
N3	0.0190 (13)	0.0278 (14)	0.0232 (14)	−0.0022 (11)	0.0052 (11)	−0.0038 (11)
N4	0.0410 (18)	0.0494 (19)	0.0326 (16)	0.0038 (15)	0.0140 (14)	−0.0011 (14)
C1	0.0262 (17)	0.0418 (19)	0.0271 (17)	−0.0081 (15)	0.0025 (14)	−0.0123 (14)
C2	0.0264 (17)	0.0359 (18)	0.0240 (15)	−0.0092 (13)	0.0084 (13)	−0.0120 (13)
C3	0.035 (2)	0.040 (2)	0.0367 (19)	−0.0128 (16)	0.0101 (16)	−0.0175 (16)
C4	0.044 (3)	0.0300 (18)	0.058 (3)	−0.0068 (16)	0.0178 (19)	−0.0173 (17)
C5	0.0286 (18)	0.0324 (18)	0.058 (3)	0.0009 (15)	0.0127 (17)	−0.0039 (17)
C6	0.0236 (16)	0.0298 (16)	0.0342 (18)	−0.0023 (13)	0.0099 (14)	−0.0017 (13)
C7	0.0210 (15)	0.0306 (16)	0.0329 (17)	−0.0041 (13)	0.0038 (13)	0.0040 (13)
C8	0.0212 (15)	0.0314 (16)	0.0252 (15)	−0.0038 (12)	0.0085 (13)	−0.0047 (12)
C9	0.0197 (15)	0.0301 (16)	0.0227 (15)	−0.0035 (12)	0.0070 (12)	−0.0020 (12)
C10	0.0209 (15)	0.0258 (15)	0.0353 (17)	−0.0023 (12)	0.0108 (13)	−0.0021 (13)
C11	0.0204 (16)	0.0307 (16)	0.0372 (18)	0.0053 (13)	0.0096 (14)	0.0094 (14)
C12	0.0188 (15)	0.0367 (17)	0.0213 (15)	−0.0003 (13)	0.0065 (12)	0.0055 (13)
C13	0.0175 (14)	0.0287 (15)	0.0232 (15)	−0.0035 (12)	0.0075 (12)	0.0000 (12)
C14	0.0174 (14)	0.0285 (15)	0.0214 (14)	−0.0007 (12)	0.0055 (12)	0.0014 (12)
C15	0.0303 (19)	0.0326 (19)	0.056 (3)	0.0001 (15)	0.0108 (17)	−0.0067 (17)
C16	0.0252 (17)	0.048 (2)	0.0294 (18)	0.0047 (15)	0.0010 (14)	0.0056 (15)
C17	0.0235 (16)	0.0283 (16)	0.0236 (15)	0.0011 (13)	−0.0007 (13)	−0.0004 (12)
C18	0.0191 (15)	0.0309 (16)	0.0255 (15)	−0.0040 (12)	0.0066 (12)	−0.0022 (12)
C19	0.051 (3)	0.149 (6)	0.051 (3)	0.012 (4)	0.006 (3)	−0.031 (4)
C20	0.065 (3)	0.079 (4)	0.056 (3)	−0.005 (3)	0.036 (3)	0.004 (3)
C21	0.043 (3)	0.043 (2)	0.038 (2)	0.0097 (17)	0.0100 (18)	0.0043 (16)
C22	0.132 (6)	0.102 (5)	0.047 (3)	−0.069 (5)	0.026 (4)	−0.016 (3)

### Geometric parameters (Å, º)

**Table:**

Pd1—Cl1	2.3084 (9)	C14—C17	1.513 (4)
Pd1—N1	2.062 (3)	O6—H18	0.840
Pd1—N2	1.942 (3)	N1—H12	0.78 (4)
Pd1—N3	2.087 (3)	N3—H13	0.72 (4)
Cl2—O1	1.434 (3)	C1—H1A	0.990
Cl2—O2	1.435 (3)	C1—H1B	0.990
Cl2—O3	1.440 (4)	C3—H2	0.950
Cl2—O4	1.433 (3)	C4—H3	0.950
O5—C21	1.231 (6)	C5—H4	0.950
O6—C22	1.304 (9)	C7—H5A	0.990
N1—C1	1.512 (5)	C7—H5B	0.990
N1—C18^i^	1.517 (5)	C8—H6A	0.990
N2—C2	1.356 (4)	C8—H6B	0.990
N2—C6	1.344 (4)	C11—H8	0.950
N3—C7	1.504 (5)	C15—H7A	0.980
N3—C8	1.522 (5)	C15—H7B	0.980
N4—C19	1.449 (6)	C15—H7C	0.980
N4—C20	1.451 (7)	C16—H9A	0.980
N4—C21	1.311 (5)	C16—H9B	0.980
C1—C2	1.497 (5)	C16—H9C	0.980
C2—C3	1.380 (5)	C17—H11A	0.980
C3—C4	1.384 (5)	C17—H11B	0.980
C4—C5	1.394 (5)	C17—H11C	0.980
C5—C6	1.374 (5)	C18—H10A	0.990
C6—C7	1.508 (5)	C18—H10B	0.990
C8—C9	1.519 (4)	C19—H14A	0.980
C9—C10	1.407 (5)	C19—H14B	0.980
C9—C14	1.422 (5)	C19—H14C	0.980
C10—C11	1.394 (5)	C20—H15A	0.980
C10—C15	1.506 (5)	C20—H15B	0.980
C11—C12	1.385 (5)	C20—H15C	0.980
C12—C13	1.415 (5)	C21—H16	0.950
C12—C16	1.509 (5)	C22—H17A	0.980
C13—C14	1.402 (4)	C22—H17B	0.980
C13—C18	1.514 (5)	C22—H17C	0.980
			
Cl1—Pd1—N1	97.43 (8)	C2—C1—H1B	109.609
Cl1—Pd1—N2	175.60 (8)	H1A—C1—H1B	108.144
Cl1—Pd1—N3	98.52 (8)	C2—C3—H2	120.657
N1—Pd1—N2	81.63 (11)	C4—C3—H2	120.648
N1—Pd1—N3	163.33 (11)	C3—C4—H3	119.486
N2—Pd1—N3	82.82 (10)	C5—C4—H3	119.487
O1—Cl2—O2	111.8 (2)	C4—C5—H4	120.689
O1—Cl2—O3	108.8 (3)	C6—C5—H4	120.678
O1—Cl2—O4	108.88 (19)	N3—C7—H5A	109.342
O2—Cl2—O3	108.95 (19)	N3—C7—H5B	109.355
O2—Cl2—O4	109.4 (2)	C6—C7—H5A	109.338
O3—Cl2—O4	109.0 (2)	C6—C7—H5B	109.347
Pd1—N1—C1	107.07 (19)	H5A—C7—H5B	107.982
Pd1—N1—C18^i^	112.50 (17)	N3—C8—H6A	109.912
C1—N1—C18^i^	109.9 (3)	N3—C8—H6B	109.900
Pd1—N2—C2	118.0 (2)	C9—C8—H6A	109.914
Pd1—N2—C6	118.3 (2)	C9—C8—H6B	109.908
C2—N2—C6	123.6 (3)	H6A—C8—H6B	108.320
Pd1—N3—C7	104.97 (18)	C10—C11—H8	118.607
Pd1—N3—C8	121.7 (2)	C12—C11—H8	118.614
C7—N3—C8	112.8 (3)	C10—C15—H7A	109.473
C19—N4—C20	117.7 (4)	C10—C15—H7B	109.469
C19—N4—C21	121.8 (4)	C10—C15—H7C	109.471
C20—N4—C21	120.5 (4)	H7A—C15—H7B	109.470
N1—C1—C2	110.2 (3)	H7A—C15—H7C	109.468
N2—C2—C1	113.8 (3)	H7B—C15—H7C	109.477
N2—C2—C3	118.8 (3)	C12—C16—H9A	109.470
C1—C2—C3	127.3 (3)	C12—C16—H9B	109.474
C2—C3—C4	118.7 (4)	C12—C16—H9C	109.465
C3—C4—C5	121.0 (4)	H9A—C16—H9B	109.474
C4—C5—C6	118.6 (4)	H9A—C16—H9C	109.469
N2—C6—C5	119.2 (3)	H9B—C16—H9C	109.475
N2—C6—C7	112.3 (3)	C14—C17—H11A	109.468
C5—C6—C7	128.4 (3)	C14—C17—H11B	109.469
N3—C7—C6	111.4 (3)	C14—C17—H11C	109.466
N3—C8—C9	108.9 (3)	H11A—C17—H11B	109.474
C8—C9—C10	121.7 (3)	H11A—C17—H11C	109.470
C8—C9—C14	118.7 (3)	H11B—C17—H11C	109.480
C10—C9—C14	119.2 (3)	N1^i^—C18—H10A	109.221
C9—C10—C11	118.9 (3)	N1^i^—C18—H10B	109.225
C9—C10—C15	124.5 (3)	C13—C18—H10A	109.220
C11—C10—C15	116.6 (3)	C13—C18—H10B	109.225
C10—C11—C12	122.8 (3)	H10A—C18—H10B	107.917
C11—C12—C13	118.4 (3)	N4—C19—H14A	109.480
C11—C12—C16	119.6 (3)	N4—C19—H14B	109.469
C13—C12—C16	122.0 (3)	N4—C19—H14C	109.473
C12—C13—C14	120.2 (3)	H14A—C19—H14B	109.474
C12—C13—C18	118.2 (3)	H14A—C19—H14C	109.472
C14—C13—C18	121.6 (3)	H14B—C19—H14C	109.459
C9—C14—C13	120.0 (3)	N4—C20—H15A	109.469
C9—C14—C17	119.5 (3)	N4—C20—H15B	109.467
C13—C14—C17	120.5 (3)	N4—C20—H15C	109.470
N1^i^—C18—C13	111.9 (3)	H15A—C20—H15B	109.476
O5—C21—N4	124.5 (4)	H15A—C20—H15C	109.470
C22—O6—H18	109.469	H15B—C20—H15C	109.476
Pd1—N1—H12	112 (3)	O5—C21—H16	117.729
C1—N1—H12	109 (3)	N4—C21—H16	117.725
C18^i^—N1—H12	106 (3)	O6—C22—H17A	109.471
Pd1—N3—H13	98 (3)	O6—C22—H17B	109.478
C7—N3—H13	106 (3)	O6—C22—H17C	109.472
C8—N3—H13	112 (4)	H17A—C22—H17B	109.469
N1—C1—H1A	109.621	H17A—C22—H17C	109.469
N1—C1—H1B	109.615	H17B—C22—H17C	109.469
C2—C1—H1A	109.615		
			
Cl1—Pd1—N1—C1	−149.86 (13)	N2—C2—C3—C4	1.9 (6)
Cl1—Pd1—N1—C18^i^	89.28 (15)	C1—C2—C3—C4	−174.0 (4)
Cl1—Pd1—N3—C7	−162.28 (13)	C2—C3—C4—C5	−2.9 (7)
Cl1—Pd1—N3—C8	68.21 (18)	C3—C4—C5—C6	0.9 (7)
N1—Pd1—N2—C2	−16.25 (19)	C4—C5—C6—N2	2.1 (6)
N1—Pd1—N2—C6	167.5 (2)	C4—C5—C6—C7	−172.7 (4)
N2—Pd1—N1—C1	25.80 (15)	N2—C6—C7—N3	31.1 (4)
N2—Pd1—N1—C18^i^	−95.06 (17)	C5—C6—C7—N3	−153.8 (4)
N2—Pd1—N3—C7	21.95 (16)	N3—C8—C9—C10	−94.4 (4)
N2—Pd1—N3—C8	−107.56 (19)	N3—C8—C9—C14	78.6 (4)
N3—Pd1—N2—C2	169.8 (2)	C8—C9—C10—C11	167.8 (3)
N3—Pd1—N2—C6	−6.50 (19)	C8—C9—C10—C15	−12.7 (6)
Pd1—N1—C1—C2	−31.6 (3)	C8—C9—C14—C13	−165.3 (3)
Pd1—N1—C18^i^—C13^i^	−66.0 (3)	C8—C9—C14—C17	15.6 (5)
C1—N1—C18^i^—C13^i^	174.8 (2)	C10—C9—C14—C13	7.9 (5)
C18^i^—N1—C1—C2	90.9 (3)	C10—C9—C14—C17	−171.2 (3)
Pd1—N2—C2—C1	1.5 (4)	C14—C9—C10—C11	−5.2 (5)
Pd1—N2—C2—C3	−174.9 (2)	C14—C9—C10—C15	174.3 (3)
Pd1—N2—C6—C5	172.8 (2)	C9—C10—C11—C12	−1.0 (6)
Pd1—N2—C6—C7	−11.6 (4)	C15—C10—C11—C12	179.5 (3)
C2—N2—C6—C5	−3.2 (5)	C10—C11—C12—C13	4.4 (6)
C2—N2—C6—C7	172.3 (3)	C10—C11—C12—C16	−175.8 (3)
C6—N2—C2—C1	177.6 (3)	C11—C12—C13—C14	−1.6 (5)
C6—N2—C2—C3	1.2 (5)	C11—C12—C13—C18	179.7 (3)
Pd1—N3—C7—C6	−33.6 (3)	C16—C12—C13—C14	178.6 (3)
Pd1—N3—C8—C9	−150.26 (17)	C16—C12—C13—C18	−0.1 (5)
C7—N3—C8—C9	83.7 (3)	C12—C13—C14—C9	−4.5 (5)
C8—N3—C7—C6	101.0 (3)	C12—C13—C14—C17	174.6 (3)
C19—N4—C21—O5	−1.8 (7)	C12—C13—C18—N1^i^	85.7 (4)
C20—N4—C21—O5	−179.0 (4)	C14—C13—C18—N1^i^	−93.0 (4)
N1—C1—C2—N2	20.9 (4)	C18—C13—C14—C9	174.2 (3)
N1—C1—C2—C3	−163.0 (3)	C18—C13—C14—C17	−6.7 (5)

Symmetry code: (i) −*x*+1, −*y*+1, −*z*.

### Hydrogen-bond geometry (Å, º)

**Table:**

*D*—H···*A*	*D*—H	H···*A*	*D*···*A*	*D*—H···*A*
O6—H18···O6^ii^	0.84	2.33	2.757 (5)	112
N1—H12···O6	0.78 (4)	2.21 (4)	2.930 (5)	153 (4)
N3—H13···Cl1^i^	0.71 (4)	2.67 (5)	3.332 (4)	156 (4)

Symmetry codes: (i) −*x*+1, −*y*+1, −*z*; (ii) −*x*+2, −*y*+1, −*z*+1.
